# Spinal Subdural Haematoma in association with anticoagulant therapy, an unusual presentation: a case report and review of literature

**DOI:** 10.1186/1757-1626-2-151

**Published:** 2009-10-12

**Authors:** Ravi Badge, Daniel Chan

**Affiliations:** 1 10 Holme Avenue, Swinley, Wigan WN1 2EH, UK; 2 The Spinal Unit, Princess Elizabeth Orthopaedic Centre, Royal Devon & Exeter Hospital, Exeter EX2 5DW, UK

## Abstract

A case of spontaneous, atraumatic subdural haematoma involving thoracic region in a 78-year-old woman on an anticoagulant therapy (Warfarin) for atrial fibrillation presented. This patient initially presented with sudden onset headache and giddiness (signs of increased intracranial pressure) followed by an acute onset neuro-deficit in lower limb. After appropriate investigations she was treated with an emergency surgical decompression of involved spinal segment. Post-operatively the patients had complete neurological recovery.

## Introduction

Spontaneous spinal subdural haematoma as a result of an anticoagulant therapy is a rare cause of spinal cord compression[1]. Subdural haematoma as a result of other causes like haematological disorders, arterio-venous malformation, meningioma and repeated attempts at lumbar punctures has been reported quite often in the literature[2].

In this report we present a patient with an anticoagulant therapy for atrial fibrillation who experienced symptoms of increased intracranial pressure and radicular symptoms related to involved spinal segment before progressing to acute spinal cord compression and thus leading to neurological deficit in lower limb.

## Case report

We report a case of spontaneous, atraumatic, spinal subdural haematoma in the thoracic region in a 78-year-old Caucasian woman of British nationality on an anticoagulant therapy (warfarin sodium) for atrial fibrillation for last five years. The patient presented to Accident &Emergency department with acute onset nausea, giddiness and headache. This was initially diagnosed as a labyrinthitis and investigated in the form of computed tomography (CT) of head which was inconclusive. The patient later developed girdle pain corresponding to 8^th ^thoracic dermatome which was interpreted as a manifestation of possibility of herpes zoster. Next day, patient developed sudden onset left lower limb weakness (Grade 2 power) and impaired coordination (positive heel to shin test) and was then urgently referred to the spinal unit for assessment.

**Figure 1:**
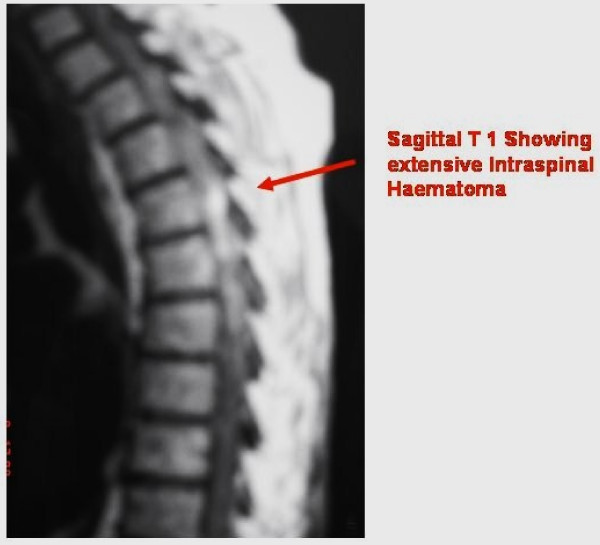
**Pre-op MRI- T1 Sagittal showing extensive intra-spinal haematoma**.

**Figure 2:**
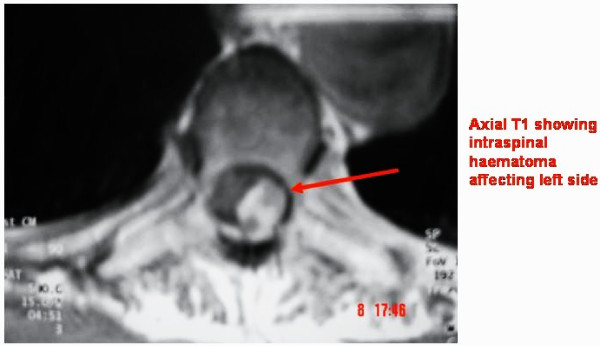
**Pre-op MRI- T1 Axial showing intra-spinal haematoma at thoracic-5 level**.

The MRI scan of the spine showed epidural haematoma extending from Thoracic 3 to 12^th ^spinal canal causing posterior compression of spinal cord (Figure 1&2).

The blood investigation on admission showed an INR of 2.7 and therefore the warfarin was stopped and Vitamin K was administered to get the INR below two. The patient was taken to the theatre for an urgent posterior decompression. Laminectomy at thoracic level five was performed with standard posterior approach.

**Figure 3:**
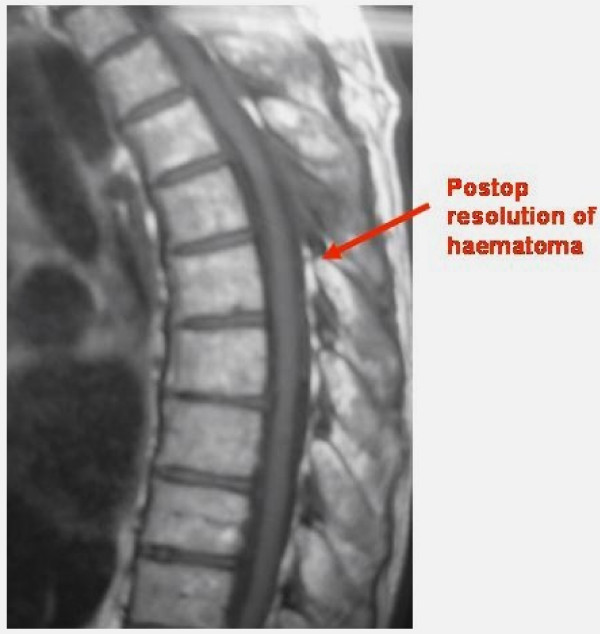
**Six month post-op MRI- T1 Sagittal showing resolution of haematoma**.

**Figure 4:**
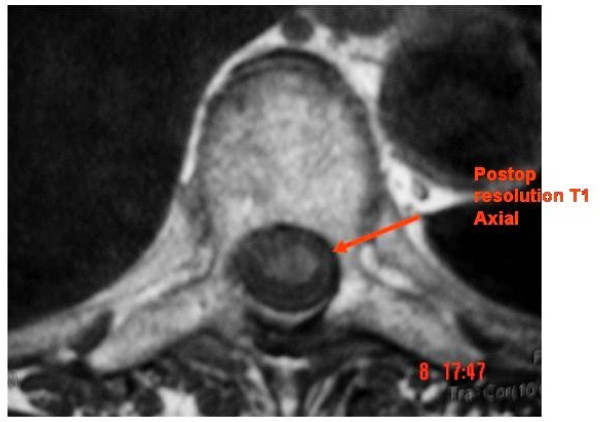
**Six month post-op MRI- T1 Axial showing resolution of haematoma**.

Intraoperatively it was found to be a sub-dural haematoma rather than an epidural as suggested by the initial MRI report. The dural layer was opened longitudinally under microscope, which revealed jelly like blood clot underneath. The blood clot was then teased off the cord using blunt dissection, which was then cleared with neural suction by intermittent irrigation & suction method. Dura closed with 6'0 nylon. The subsequent pathological report confirmed the diagnosis of haematoma. Post-operatively patient had significant neurological recovery with Grade 4 power in lower limb and improvement in lower limb coordination. The patient had repeat MRI scan of thoracic spine six months after the decompression, which confirmed the regression of subdural haematoma (Figure 3&4).

## Discussion

Spontaneous intracranial haemorrhage is reported more frequently than intra-spinal in patients undergoing anticoagulation therapy. By far the most common form of intra-spinal haemorrhage occurs in the epidural space. Although sub-dural haematoma is well recognised, it's still a rare and can be potentially serious clinical entity[3].

The typical clinical presentation is sudden onset severe back pain with or without radicular symptoms. Intra spinal haematoma is the first diagnosis to be considered in patients on anticoagulant therapy presenting with signs of acute cord or cauda-equina compression[4,5].

It may present with the misleading clinical picture of increased intracranial pressure as described in this reported case. The symptoms of cord compression may progress over a variable course to cause major neurological deficit, usually paraplegia with bowel and bladder involvement. In our case even the clinical examination on initial presentation didn't give a hint regarding the nature of lesion, it is therefore advisable to assess the patients on anticoagulation therapy thoroughly for possibility of an intra-spinal haematoma in order to avoid any major neurological complications.

According to literature, MRI is the method of choice to diagnose SSDH. It also allows surgical planning with regards to cranio-caudal extension and dorsoventral location of the haematoma. On sagittal images SSDH appear clumped and loculated with concave delineation, conversely epidural haematoma has a convex shape. The CT scan may be more helpful in compartmentalising the haematoma. Spontaneous onset spinal subdural haematoma in patients with anticoagulation therapy is a neurological emergency therefore early diagnosis, discontinuation of anticoagulant and urgent surgical decompression is recommended to allow neurological recovery[1,2,4,6,7].

## Conclusion

Spinal subdural haematoma is a neurological emergency. The anticoagulants should be stopped immediately and appropriate investigations should be carried out to localise the pathology. It may be helpful to have thorough investigation of the entire spinal column along with brain in patients presenting with signs of increased intracranial pressure.

Although there are reported cases with spontaneous recovery, an urgent surgical intervention will result in a greater likelihood of neurological recovery.

## Consent

Written informed consent was obtained from the patient for publication of this case report and accompanying images. A copy of the written consent is available for review from the journal's Editor-in-Chief

## Competing interests

The authors declare that they have no competing interests.

## Authors' contributions

RB performed the literature search and wrote the draft of the manuscript; DC read and approved the final manuscript.
